# Supplementary material for: Enhancement of Nutritional Substance, Trace Elements, and Pigments in Waxy Maize Grains through Foliar Application of Selenite
Source: Foods. 2024 Apr 26;13(9):1337. doi: 10.3390/foods13091337 (PMC11083303; doi:10.3390/foods13091337)
Supplement: Supplementary file 1 [file foods-13-01337-s001.zip › foods-2951261-supplementary.pdf]

Table 1. The correlation matrix eigenvalues and corresponding matrix eigenvectors of nutritional quality, trace elements and pigment content traits of maize kernels in 2019

Primary Component		Principal Component Number					
		1	2	3	4	5	6
Eigenvalue		6.645	3.562	1.968	1.214	0.754	0.475
Percentage of variance (%)		41.532	22.260	12.299	7.586	4.714	2.969
Cumulative (%)		41.532	63.792	76.090	83.676	88.391	91.360
Load factor	Soluble sugar	-0.281	0.328	0.029	0.141	0.146	-0.088
	Sucrose	-0.279	0.316	0.087	0.245	-0.029	-0.002
	Vitamin C	0.332	0.014	0.265	0.008	-0.143	-0.087
	Protein	0.301	0.176	0.239	0.108	0.032	-0.122
	Lysine	0.132	0.260	0.063	-0.221	0.822	0.166
	Fat	0.183	0.216	-0.335	0.216	-0.289	0.691
	Total starch	0.063	0.458	-0.009	-0.372	-0.202	-0.085
	Amylose	0.314	0.112	-0.150	-0.220	0.100	0.190
	Amylopectin	0.032	0.463	0.007	-0.362	-0.220	-0.108
	Anthocyanin	0.317	-0.117	0.262	0.021	-0.005	0.284
	Carotenoid	-0.268	0.338	-0.082	0.207	-0.035	0.142
	Fe	0.361	0.007	0.011	0.058	-0.036	0.040
	Mn	0.259	0.066	-0.360	0.308	0.093	-0.349
	Cu	0.217	0.133	-0.385	0.393	0.193	-0.194
	Zn	0.263	0.209	0.246	0.178	-0.205	-0.319
	Se	-0.043	0.130	0.558	0.410	0.092	0.215

Table 2. The correlation matrix eigenvalues and corresponding matrix eigenvectors of nutritional quality, trace elements and pigment content traits of maize kernels in 2020

Primary Component		Principal Component Number					
		1	2	3	4	5	6
Eigenvalue		6.808	4.051	2.137	1.024	0.541	0.511
Percentage of variance (%)		42.551	25.321	13.359	6.402	3.382	3.195
Cumulative (%)		42.551	67.872	81.231	87.633	91.015	94.210
Load factor	Soluble sugar	-0.363	0.120	0.074	0.072	0.137	0.049
	Sucrose	-0.342	0.165	0.104	0.134	0.123	0.061
	Vitamin C	0.304	0.178	-0.252	0.175	0.145	0.081
	Protein	0.002	0.387	0.107	0.060	0.152	-0.790
	Lysine	0.337	0.166	-0.061	-0.104	0.067	0.067
	Fat	0.081	0.319	0.228	0.284	-0.790	0.041
	Total starch	0.052	0.419	0.111	-0.457	0.072	0.191
	Amylose	-0.324	0.185	0.184	-0.049	0.001	0.150
	Amylopectin	0.107	0.403	0.084	-0.464	0.074	0.173
	Anthocyanin	0.343	0.098	-0.173	0.144	0.082	-0.085
	Carotenoid	-0.283	0.277	0.104	0.155	0.002	-0.106
	Fe	0.205	0.141	0.295	0.493	0.171	0.389
	Mn	0.142	-0.151	0.526	-0.132	0.081	0.120

**Cu**	0.164	-0.127	0.520	0.163	0.379	-0.135
**Zn**	0.339	0.172	0.022	0.040	-0.054	-0.102
**Se**	-0.132	0.317	-0.352	0.303	0.306	0.234

Table 3. The principal component scores of nutritional quality, trace elements and pigment content of maize grains in 2019.

2019	PC1	PC2	PC3	PC4	PC5	PC6	PC	Ranking
**JS0**	0.201	-1.682	-0.142	-0.688	-0.277	0.764	-0.351	8
**JS1**	0.797	-0.742	-0.673	-0.161	0.101	-0.787	0.052	6
**JS2**	1.291	-0.075	-1.300	0.882	0.745	0.045	0.463	2
**JS3**	1.445	1.169	0.703	-1.365	-0.056	0.561	0.857	1
**JS4**	0.592	-0.755	2.038	0.939	-0.494	-0.323	0.367	3
**CS0**	-1.455	-0.692	-0.558	-1.136	0.072	-0.194	-0.916	10
**CS1**	-0.810	0.226	-0.400	-0.335	-0.473	-0.387	-0.394	9
**CS2**	-0.209	1.077	-0.850	0.766	-0.434	-0.267	0.078	5
**CS3**	-0.604	1.310	0.259	0.411	0.162	-0.369	0.101	4
**CS4**	-1.248	0.164	0.923	0.687	0.655	0.957	-0.257	7

Table 4. The principal component scores of nutritional quality, trace elements and pigment content of maize grains in 2020.

2020	PC1	PC2	PC3	PC4	PC5	PC6	PC	Ranking
**JS0**	0.726	-1.131	-0.945	-0.776	-0.639	-0.551	-0.192	7
**JS1**	0.944	-0.928	0.446	0.170	0.436	0.407	0.265	4
**JS2**	1.146	-0.299	0.970	1.005	0.088	-0.096	0.606	2
**JS3**	1.127	1.542	0.363	-1.586	-0.717	0.018	0.793	1
**JS4**	0.864	0.615	-1.589	1.204	0.628	0.045	0.411	3
**CS0**	-1.092	-1.236	-0.342	-0.571	-0.868	-0.153	-0.894	10
**CS1**	-0.847	-0.692	0.587	-0.308	0.331	0.080	-0.463	9
**CS2**	-0.690	0.056	1.181	0.471	0.802	0.559	-0.047	6
**CS3**	-0.954	1.255	0.757	0.114	-0.246	-1.170	-0.025	5
**CS4**	-1.224	0.817	-1.429	0.276	0.185	0.861	-0.453	8
